# A pancreas tumor derived organoid study: from drug screen to precision medicine

**DOI:** 10.1186/s12935-021-02044-1

**Published:** 2021-07-27

**Authors:** Jia Yao, Man Yang, Lawrence Atteh, Pinyan Liu, Yongcui Mao, Wenbo Meng, Xun Li

**Affiliations:** 1 Key Laboratory of Biological Therapy and Regenerative Medicine Transformation of Gansu Province, The First Hospital of Lanzhou University, Lanzhou, Gansu China; 2 The First Clinical Medical College of Lanzhou University, Lanzhou, Gansu China; 3 Department of General Surgery, The First Hospital of Lanzhou University, The First Clinical Medical School of Lanzhou University, Lanzhou, 730000 Gansu China

**Keywords:** Pancreas cancer, Pancreatic ductal adenocarcinoma (PDAC), Pancreas tumor derived organoids (PTOs), Drug screen, Precision medicine

## Abstract

Pancreatic ductal adenocarcinoma (PDAC) one of the deadliest malignant tumor. Despite considerable progress in pancreatic cancer treatment in the past 10 years, PDAC mortality has shown no appreciable change, and systemic therapies for PDAC generally lack efficacy. Thus, developing biomarkers for treatment guidance is urgently required. This review focuses on pancreatic tumor organoids (PTOs), which can mimic the characteristics of the original tumor in vitro. As a powerful tool with several applications, PTOs represent a new strategy for targeted therapy in pancreatic cancer and contribute to the advancement of the field of personalized medicine.

## Introduction

Pancreatic ductal adenocarcinoma (PDAC), an exocrine pancreatic malignancy, is a rapidly developing and fatal disease that accounts for the majority of pancreatic cancers. It is the 8th and 9th deadliest malignancy in males and females globally, respectively [1]. Although PDAC treatments, particularly immunotherapy and adjuvant chemotherapy, have been optimized, 5-year survival in patients with PDAC remains low at 7–8% [2]. There are multiple factors responsible for such suboptimal results, e.g., late diagnosis, quick progression featuring metastasis, and resistance to currently available chemotherapeutics. Unfortunately, the majority of patients are diagnosed at later phases after metastasis is involved, and such individuals have already been affected for 6–12 months prior to diagnosis. Accordingly, gaining insights into the mechanisms of disease initiation and progression is of vital importance for early detection and risk stratification, and could aid in developing targeted therapeutic strategies [3, 4]. Despite the progress made with respect to insights into the mechanisms of PDAC pathogenesis, the actual impact in terms of benefits to patients remains unclear [5, 6]. Thus, novel model systems have been proposed and adopted to address the abovementioned issue with the hope that data could be translated into optimized diagnostics and therapeutics [7]. The present study highlights a patient-derived pancreatic tumor-derived organoid (PTO), which could combine drug and genomic/proteomic screening in vitro, thus raising the hope of precision therapy for pancreatic cancer [8, 9].

## Pancreatic tumor organoid culture system

### 3D cell culture model

The 3D cell culture model is a method avoiding cell attachment to the plate by growing suspension or matrix-embedded cells. The first attempts to develop cancerous pancreatic cells spheroids have failed owing to limited cell viability and longevity [10]. Spheroids that are excessively small result in the loss of cells due to the shear stress on cells in low adhesion cultures. Spheroids that are big will affect the diffusion of oxygen and metabolism of substances in cells within spheroids, resulting in the inconsistent differentiation of the whole spheroid [11]. However, recently, 3D cultures of murine and human pancreatic cells have been successfully established in multiple laboratories using special matrices that help maintain interactions among cells and between cells and the matrix, promoting spheroid structures [12, 13].

Pancreatic spheres, which could be created from pancreatic ductal and acinar cells, are likely to simulate some PDAC features in vivo, including microenvironmental parameters and drug response [14–16]. 3D spheres based on embryonic pancreatic cells partly reflect pancreas development, and express *PDX1* and *SOX9* [17, 18]. Ductal cell-derived spheres have been utilized for evaluating pancreatic carcinogenesis, notably the function of *KRAS* mutation and in drug assessment [19–22].

The initial complete protocol for directly purifying ductal epithelial cells from the mouse pancreas as well as duct-like cells, which does not require further culture steps for 3D culture, was reported in 2013 [23]. This technique utilizes Dolichos biflorus agglutinin (DBA) in magnetic bead purification. Ductal cells could be grown on and inside a collagen matrix for 2D and 3D cultures, respectively [24]. Notably, the above method could be applied in several pathological states, such as inflammation and cancer, and physiological processes, such as embryonic development [25].

### Organoid culture model

3D-culture spheres have inspired a novel ex vivo model called “tumor organoids”. This involves cells cultured in a 3D structure directly from primary tissue specimens or cancer cell lines capable of self-renewal and self-organization, with appearance and functional properties comparable to those of the source tissue [26–28]. Tumor organoids could be indefinitely passaged with preserved genetic properties, like cell epigenetic markers, functional characteristics, etc. They also share numerous features with 3D spheres. However, 3D cultures are obtained from monolayer cells, while tumor organoids are generated from tissue specimens in 3D cultures using the same protocol described previously by our group [29].

Briefly, tumor organoids are formed by digesting (enzymatically or mechanically) original tumor tissues, which undergo embedding in a matrix (collagen or Matrigel) [30]. Additionally, particular growth factors and differentiation regulators, including epidermal growth factor (EGF), fibroblast growth factor 10, Rspo1 (Wnt pathway inducer), Noggin (BMP pathway suppressor), Wnt3a, nicotinamide, *N*-acetylcysteine, gastrin, and A83-01 (Alk suppressor), are needed to supply mesenchymal-based signals [31]. Furthermore, normal (untransformed) human tumor organoids developed from ductal cells or tumor tissue samples require supplementation with prostaglandin E2. Flow cytometry and magnetic beads (with linked DBA) are optimal for isolating ductal cells, although non-ductal cells have been shown to be not feasible and are thus eliminated after one passage [32, 33].

The tumor organoid culture system for pancreatic tissues was first described in 2013 [34]. Subsequently, an organoid model derived from mouse and human adenocarcinoma of the pancreas has been successfully established by embedding cells in Matrigel [26]. Researchers have used serum-free media supplemented with multiple growth factors for propagating mouse adult pancreatic duct cells. Such media activate Wnt signaling, expanding ductal structures in serum-free conditions, further upregulating Lgr5 (stem cell biomarker and RSPO1 receptor) and promoting self-renewal [34]. Additional vital constituents of these media include Glutamax, HEPES, Noggin, Gastrin I, nicotinamide, EGF, fibroblast growth factor 10, *N*-acetylcysteine, and B27 supplement, and in human specimens, Wnt3a and Primocin [35]. Remarkably, the tumor organoids generated are physiologically similar to the original pancreatic tumor tissues. In addition, they have ductal epithelial cell biomarkers but no genes reflecting acinar and endocrine lineages. After tumor organoids were orthotopically transplanted into immunodeficient mouse models, pre-invasive tumors likening preneoplastic lesions (PanIN) that progressed to invasive adenocarcinoma and metastasize were detected. Therefore, this represents an attractive model for cancer progression.

Furthermore, murine PTOs have been subjected to gene expression analysis (RNAseq) and proteomics (mass spectrometry), revealing gene and proteomic profiles are related to pancreatic cancer progression. In another method, fibroblasts and tumor cells could be propagated in Matrigel and complete medium (1:2). The latter medium contained 10% fetal bovine serum, 1% penicillin and streptomycin cocktail, and 10 ng/mL EGF receptor. Such conditions facilitate the generation of tumor cells with fibroblasts from human and mouse PDAC [25, 30].

A method exploiting an air–liquid interface that encompasses inner collagen gel-packed cells directly exposed to air (cells in contact with elevated oxygen amounts) has been developed [37]. This technique utilizes a matrix containing collagen I in lieu of Matrigel [38] and allows 3D organoid culture from newborn or adult mouse tissue samples without exogenously supplementing growth factors.

Another strategy to develop PTOs from pancreatic cancer cell lines, included endothelial or mesenchymal cells, involves the self-assembly process [39–41]. Cancer-associated fibroblasts participate in extracellular matrix (ECM) production and contribute to tumor growth and resistance to chemotherapeutics. Lately, a research group developed a co-culture model of pancreatic cancer organoids and stellate cells [31, 36]. This model yielded increased proliferation degree ofpancreatic cancer cells [42]. Furthermore, the authors reported that cancer-related fibroblasts were heterogeneous, expressing a range of levels of smooth muscle actin and interleukin (IL)-6 based on their proximity to the organoids. The above findings indicate that ECM played a important role in pancreatic cancer cells proliferation and differentiation [13, 43].

**Fig. 1:**
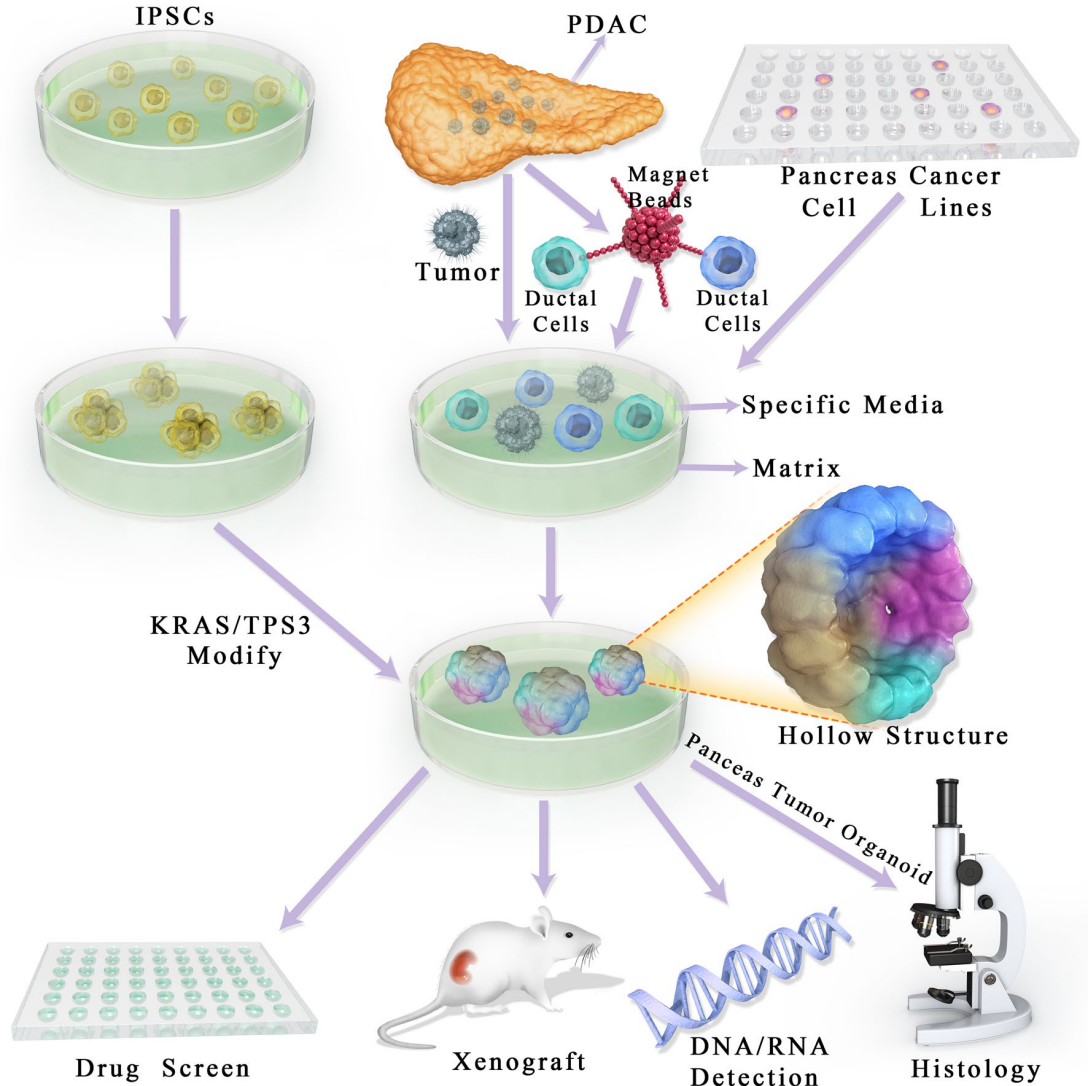
Induction of pancreas tumor organoid

Despite the easy access to cell/tissue resources for PTOs establishment, the interactions among different cell types and between the cells and the stroma remain unclear. PTOs can be generated not only by excising tissues or biopsy, but also from endoscopy-mediated fine-needle aspiration or biopsy specimens. Moreover, they could be produced from small tissue quantities [44–46]. Lastly, PTOs retain the genetic phenotypes and biological features of the original tissue. In addition, researchers also found that PTOs could be generated from iPSCs with Kras/tps3 modification [22]. The primary culture model of PTOs is depicted in Fig. 1. Well-established PTO culture systems are summarized in Table 1.

**Table 1:** Pancreas tumor organoids culture system established for drug and biomarker screen

Tissue	Species	Matrix	Trans-plantation	Drug testing	Biomarker screen	References
Nomal pancreas PanIN PDAC	Murine Human	Embedded in Matrigel	Generation of PDAC and PanIN	Yes	Yes	[9] [24] [27, 28] [30]
iPSCs PDAC	Human	Media and Matrigel	Generation primary tumor	Yes	Yes	[25]
PDAC	Murine Human	Media and Matrigel	–	Yes	Yes	[34] [89]
Neonatal tissue PDAC iPSCs	Murine Human	Air-liquid interface on Matrigel	Generation of PDAC	Yes	Yes	[32]

*PanIN* pancreas intraepithelial neoplasia, *PDAC* pancreas ductal adenocarcinoma, *iPSCs* induced pluripotent stem cells

## Applications of PTOs

**Fig. 2:**
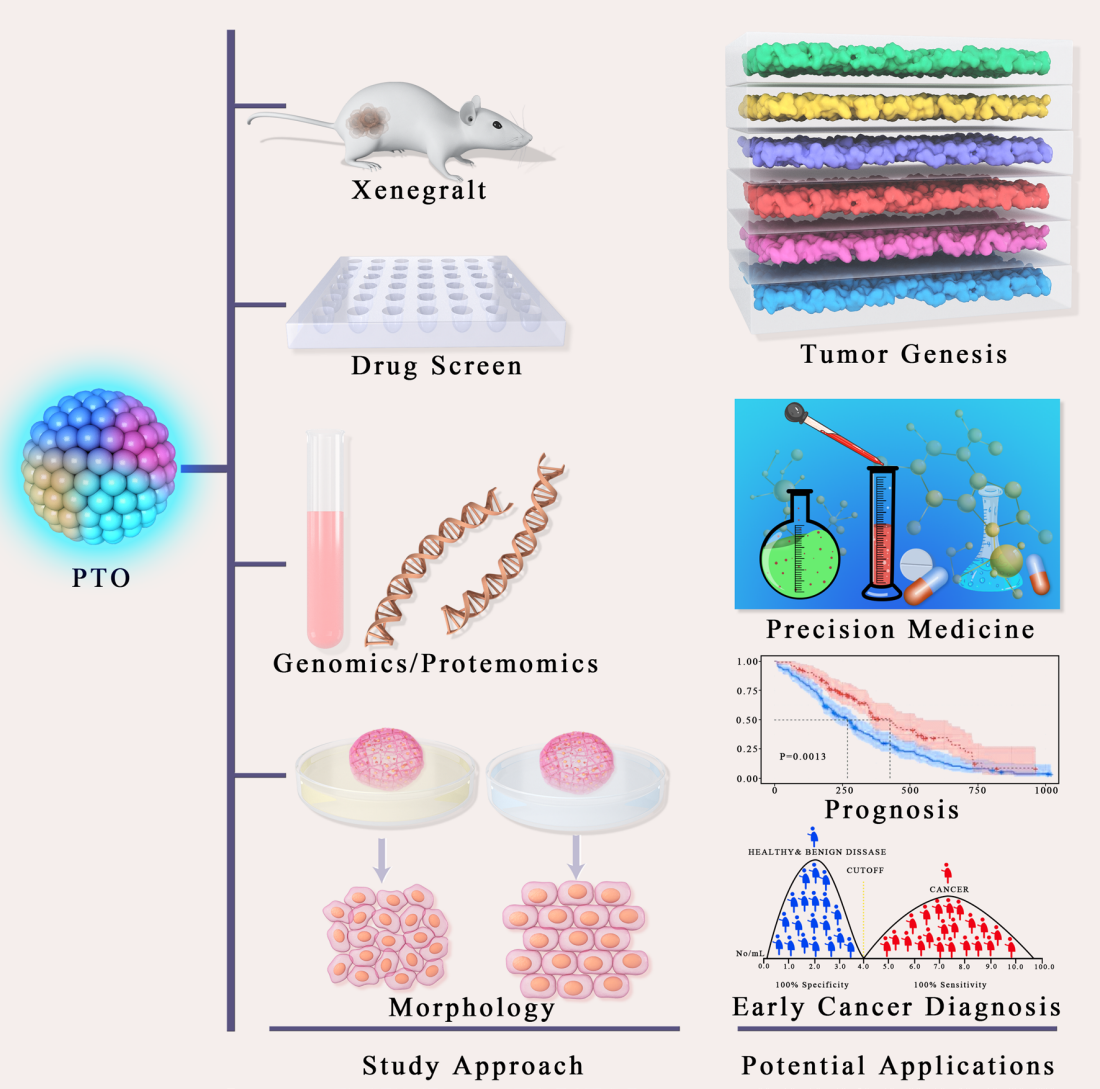
Potential applications of pancreas tumor organoid

PTOs are an effective research tool that can be utilized in numerous major areas of pancreatic tissue pathology [47]. PTOs can be obtained quickly and do not require large tissue amounts, thus enabling for drug development and the assessment of biomarkers for diagnosis. Various disease phases and clinical scenarios could also be mimicked by such a tool. Large genotranscriptomic trials of human pancreatic malignancies have assessed surgical samples. However, only 20% of pancreatic cancer cases are eligible for surgical resection, with the remaining 80% showing advanced disease and poor prognosis [48–51]. The aforementioned shortcomings can be potentially resolved using the PTO platform [52, 53] (Fig. 2).

### Neoplasia modeling

PTOs allow the modeling of human pancreatic cancer development in vitro [47] and thus represent an excellent approach for studying pancreatic cancer progression. Because PTOs can routinely be obtained from normal human epithelia, mutational processes during different phases of malignancy can be monitored in vitro, and in vitro culture of a range of premalignant pancreas neoplasias is now feasible [27]. However, PTOs do not rely on the R-spondin protein to activate Wnt pathway mutations, and their dependence on other niche growth factors is specifically lost in the adenoma-to-carcinoma transition [32, 45, 54]. Another relevant application of PTOs is their use in xenografts [55, 56]. Upon PTO transplantation into immunodeficient mice, PanINs capable of developing invasive adenocarcinoma and metastasis are generated, thereby representing an efficient and convenient option for studying tumor progression and identifying novel biomarkers in the initial phases of PDAC [57–59].

### Biomarker identification

For identifying biomarkers and stratifying patients according to genetic profile and therapeutic response, biobanks of 3D organoids attract increasing attention. Organoid biobanks produced from tissues specimens collected perioperatively or by endoscopic ultrasound biopsy allow the sampling of a broad range of tumors of various stages [39, 48, 60–65]. Interestingly, PTOs generated from frozen tumor tissues exhibit comparable morphology, viability, and metabolism to those derived from fresh tissues [66]. These findings indicate that pancreatic cancer-specific and early-phase biomarkers can be feasibly identified [30, 67]. mRNA expression analysis of human pancreas organoid reveals that hPOs express increased levels of the adult stem cell marker *LGR5*. Organoid developed from isolated ducts and islets all express similar levels of the pancreatic progenitor and beta-cell marker *PDX1* [68]. It was also reported that ducts-derived organoid express higher levels of ductal marker *SOX9* in comparison to islets. These findings suggest pancreas organoid maintain a pancreatic ductal identity during in vitro culture [57].

### Genomic studies

PTOs constitute a new tool for analyzing gene expression in tumor cells, with high selectivity [69, 70]. They could be utilized for validating genetic alterations involved in cancer progression and identifying genes related to different phases of tumor progression, therapeutic response, and prognosis [54, 60, 62, 63, 71–73]. For instance, an organoid model system was used for evaluating *NRF2*’s role in PDAC progression and knocking down its transcription factor in human and mouse organoids [74]. The authors revealed low proliferation in human tumor-like organoids not expressing *NRF2*. In addition, these authors demonstrated an association between *NRF2* and mRNA translation through REDOX regulation [36, 51, 67, 75–89].

PTOs is not retained well when assessing structural variation events, but there are striking cases of clustering of SV events across particular chromosomes that are retained when tumors are implanted into their respective disease models [13]. Comparison of tumors, PDXs, and PTOs revealed that several genetic aberrations are sample-specific, PDXs and PDOs may serve as tractable and transplantable systems for probing the molecular properties of PDAC [90].

### Tumor organoid biobanks

Most specimens assessed by cancer consortia, including the International Cancer Genome Consortium and The Cancer Genome Atlas, are perioperatively obtained samples of primary tumors, while metastatic tumors usually reflect the lethal phase of cancer. In theory, PTOs enable the expansion of limited tumor specimens, thereby allowing the assessment of malignant cells at all stages [61]. PTO biobanks broaden the patient sample types that can be studied in the laboratory. Biobank research has primarily verified that PTOs have the features of respective primary tumors, at least according to the data obtained from bulk DNA sequencing. Nevertheless, whether intratumoral heterogeneity is observed in organoid cultures remains unclear [29]. An additional unstudied issue is the clonal drift of “bulk” organoids in cultures maintained for extended periods.

Several initiatives have been implemented to increase the availability of well-characterized PTO biobanks in the academia and industry. The nonprofit HUB (www.hub4organoids.eu) provides established organoid biobanks. The Human Cancer Models Initiative (https://ocg.cancer.gov/programs/HCMI) represents a collaborative international consortium building cancer-derived culture models matched to genomic findings and patient features [91]. The HCMI’s objective is to improve the availability of the built models and relevant information as a community resource [53, 61]. Compounded with the technical issues of banking living materials, ethical problems and informed consent challenges associated with such biobanks are complicated.

### From drug screening to precision medicine

PTOs constitute a tool for rapid drug assessment of individual tumors before or in parallel to implementing treatment in patients with PDAC [92, 93]. Although a one-week time period between biopsy and drug selection has been reported, more recent studies have suggested that extensive drug screening should be performed within 3–4 weeks post-biopsy [45, 60, 94]. This technique can potentially reveal individual treatment vulnerabilities according to the genetic mutation profile and therapeutic response in organoids or determine the next lines of therapy in case of ineffective first-line treatment [95, 96]. It is also noteworthy that tumors derived from KPC and KC mouse models are heavily used for organoid development and drug screening, which provided a proven platform for drug screen for pre-clinical research [31, 97].

**Fig. 3:**
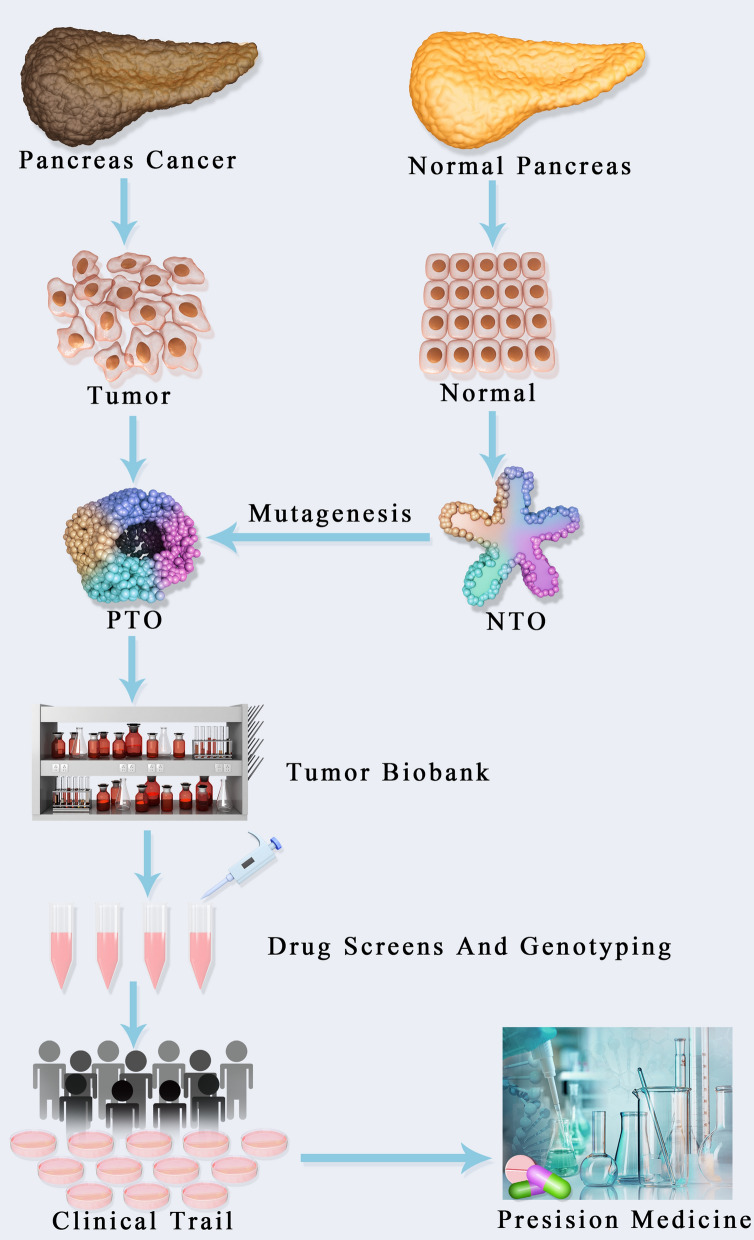
Presicion medicine based on pancreas organoid technology

Additionally, drug screening could be examined in combination with dynamic live imaging for obtaining functional optical metabolic findings [45, 63, 94, 98–102] (Fig. 3). The above multiphoton microscopy method could help in detecting cell metabolism alterations by measuring auto-fluorescence intensity as well as the half-lives of reduced nicotinamide (NAD) and flavin (FAD) adenine dinucleotides. In addition, it can detect heterogeneity, identify nonresponsive subclones, and differentiate between pre-malignant and invasive lesions [103, 104]. Optical metabolic imaging is highly sensitive in revealing metabolic alterations 1–2 h following treatment with effective drugs, and such changes correlate with the expected response (i.e., *HER2* expression in breast cancer) [105]. Further, optical metabolic imaging distinguishes cell types and drug response [18, 29, 41, 62, 63, 106, 107]. For instance, fibroblasts from PDAC organoids show drug response, despite no overt cell death enhancement [94]. Thus, this method could be adopted to evaluate the response of PDAC patients to stroma-targeting therapies in tumor organoids [23, 108–110].

Owing to the poor prognosis of individuals with metastatic pancreatic cancer, precision therapy for pancreatic cancer remains challenging [82]. In several cases, PTO pharmacotyping was completed in less than 4 weeks, demonstrating the potential of PTOs to determine the best treatment in a clinically meaningful time period in early and late stages of pancreatic cancer [111]. Because complementary genomic and transcriptome analysis is feasible in patients with advanced pancreatic cancer, PTO drug typing and transcriptome characteristics can be prospectively validated, even when first-line therapy is applied [30, 52, 85].

**Fig. 4:**
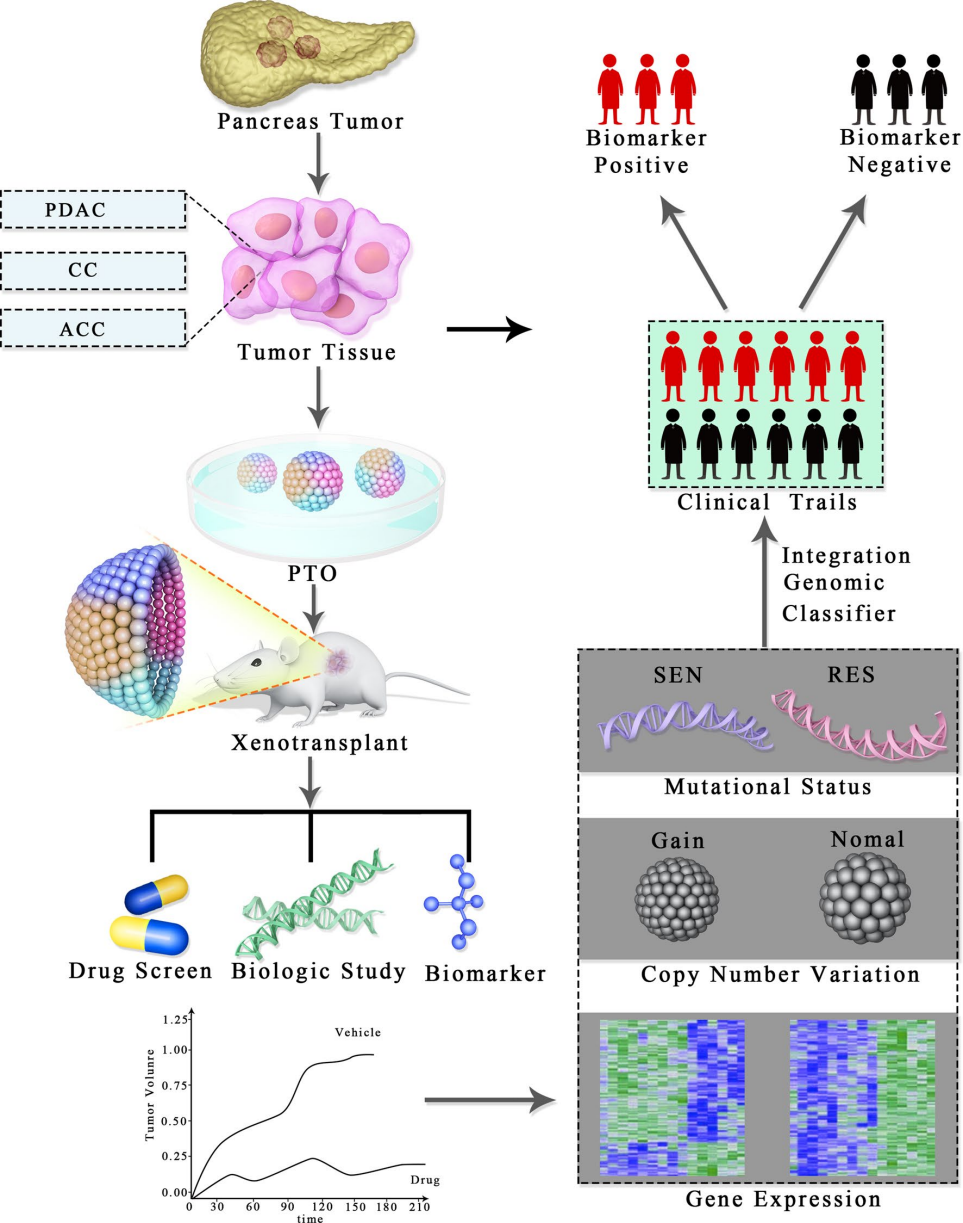
Pancreas tumor organoid xenotransplant—from surgery to clinical trails

However, this methodology is not uniformly successful in all PDAC patients [53]. Chemosensitivity profiles might stratify and thus ameliorate the initial patient care in pancreatic cancer. Moreover, in combination with longitudinal PTOs’ molecular and pharmacologic assessments, such techniques could be modified for optimizing individual patient care [63] (Fig. 4).

## Challenge and future directions

Although PTO systems and their potential applications have attracted increasing attention, their high cost and time-consuming nature cannot be overlooked. In addition, PTOs lack numerous constituents, including fibroblasts, and endothelial, immune and neural cells [39], which result in PTOs developing is the loss of vascularity and immune cell proportion during PTOs subculture. To address these limitations, studies focused on co-culture of organoids with other cell types for generating a more “physiological” microenvironment and identifying putative cell–cell interactions are underway [48].

Although the application of PTOs in pancreatic cancer is at its early stage, many studies have demonstrated several advantages, including the ease of drug testing, the predictive value on PDAC’s early diagnosis, and the stability of features shared with the original tumor. Ongoing clinical studies are evaluating the potential utilization of PTOs as a platform for pre- and post-therapy. Standardizing protocols for PTO production is also required for reproducibility. Ideally, optimization should encompass the expansion of PTO development techniques to other pancreatic lesion types, including pancreatic cystic lesions (mucinous cystic and intra-papillary mucinous neoplasms) and neuroendocrine lesions. Moreover, cheaper culture materials must be identified.

## Conclusion

Various animal models of PDAC have been established, with each approach contributing to the assessment of PDAC’s pathogenetic mechanisms. Overall, PTOs constitute a promising and effective tool for tumor targeted therapy, and could contribute to the application of precision medicine in pancreatic cancer.

## Data Availability

Not applicable.
